# The Human Host Defense Peptide LL-37 Interacts with *Neisseria meningitidis* Capsular Polysaccharides and Inhibits Inflammatory Mediators Release

**DOI:** 10.1371/journal.pone.0013627

**Published:** 2010-10-26

**Authors:** Susu M. Zughaier, Pavel Svoboda, Jan Pohl, David S. Stephens, William M. Shafer

**Affiliations:** 1 Division of Infectious Diseases, Department of Medicine, Emory University School of Medicine and Laboratories of Microbial Pathogenesis, Atlanta, Georgia, United States of America; 2 Microchemical and Proteomics Facility, Emory University School of Medicine and Laboratories of Microbial Pathogenesis, Atlanta, Georgia, United States of America; 3 Department of Microbiology and Immunology, Emory University School of Medicine and Laboratories of Microbial Pathogenesis, Atlanta, Georgia, United States of America; 4 Department of Veterans Affairs Medical Center, Atlanta, Georgia, United States of America; New York University, United States of America

## Abstract

Capsular polysaccharides (CPS) are a major virulence factor in meningococcal infections and form the basis for serogroup designation and protective vaccines. Our work has identified meningococcal CPS as a pro-inflammatory ligand that functions through TLR2 and TLR4-MD2-dependent activation. We hypothesized that human cationic host defense peptides interact with CPS and influence its biologic activity. Accordingly, the interaction of meningococcal CPS with the human-derived cationic peptide LL-37, which is expressed by phagocytic and epithelial cells that interface with meningococci during infection, was investigated. LL-37 neutralized the pro-inflammatory activity of endotoxin-free CPS as assessed by TLR2 and TLR4-MD-2-dependent release of TNFα, IL-6 and IL-8 from human and murine macrophages. The cationic and hydrophobic properties of LL-37 were crucial for this inhibition, which was due to binding of LL-37 to CPS. LL-37 also inhibited the ability of meningococcal CPS to induce nitric oxide release, as well as TNFα and CXCL10 (IP-10) release from TLR4-sufficient and TLR4-deficient murine macrophages. Truncated LL-37 analogs, especially those that retained the antibacterial domain, inhibited vaccine grade CPS and meningococcal CPS prepared from the major serogroups (A, B C, Y and W135). Thus, LL-37 interaction with CPS was independent of specific glucan structure. We conclude that the capacity of meningococcal CPS to activate macrophages via TLR2 and TLR4-MD-2 can be inhibited by the human cationic host defense peptide LL-37 and propose that this impacts CPS-based vaccine responses.

## Introduction

*Neisseria meningitidis* is a strict human pathogen that can cause both meningitis and fulminant septicemia. Frequently, disease occurs in epidemics with substantial mortality such as been observed in the sub-Saharan region of Africa (e.g., the so-called “meningitis belt”) [1]. Prevention of meningococcal disease has been a major public health effort for several years and the capsular polysaccharides (CPS) produced by meningococci have served as the basis for past and current vaccines. The CPS produced by *N. meningitidis* are considered as major virulence factors because they protect the bacteria from complement-mediated killing, as well inhibiting phagocytosis by professional phagocytes [2], [3]. The different CPS structures expressed by *N. meningitidis* form the basis for serogroup designation and protective vaccines [1], [4].

Innate immune host responses are rapidly initiated following the recognition of microbial molecules such as endotoxin, peptidoglycan, lipoteichoiec acid, lipopeptides, zymosan and nucleic acids expressing pathogen associated molecular patterns (PAMPs) or endogenous “danger” molecules (DAMPS) release by damaged cells [5], [6]. The outcome of host-pathogen interactions frequently leads to the release of pro-inflammatory mediators such as cytokines, chemokines, eicosanoids, reactive oxygen species (ROS) and host defense peptides. Host defense peptides have been shown to possess antimicrobial and immunomodulatory activities [7]. In this respect, we have shown that the sole human cathelicidin termed LL-37 not only has anti-meningococcal activity [8], but also interacts with meningococcal lipooligosaccharide (LOS) and as a consequence, inhibits release of pro-inflammatory mediators that is normally driven via a TLR4/MD2 receptor complex [9].

Recently, CPS from *Bacteroidis fragilis* [10] and *Streptococcus suis* [11] were shown to be recognized by Toll-like receptor 2 (TLR2). Based on these observations, we questioned whether meningococcal CPS could modulate TLR-mediated responses and if such responses are subject to regulation by host defense peptides. We found that preparations of meningococcal CPS induce the release of pro-inflammatory mediators from human and murine macrophages via TLR2 and TLR4/MD-2 (S. Zughaier, unpublished observations, and see below). Accordingly, the pro-inflammatory properties of meningococcal CPS and modulation by host defense peptides were investigated. LL-37 was used as a model peptide because it has been invoked as an immune modulator [7]. Understanding this interplay between CPS and LL-37 in controlling innate immune responses is important because possession of CPS is essential for invasive meningococcal disease and intracellular survival [3], [12]. Additionally, sublethal levels of LL-37, which are likely to occur *in vivo*, have been shown to cause increased expression of meningococcal genes involved in CPS biosynthesis [13]. Because LL-37 and other AMPs participate in both clearance of invading pathogens and detoxification of bacterial ligands (e.g., endotoxin and lipoteichoic acid) possessing pro-inflammatory properties, the ability of LL-37 and its synthetic analogs to block CPS-mediated pro-inflammatory responses was determined. Using vaccine-grade and endotoxin-free CPS, the host defense peptide LL-37 was found to interact with meningococcal CPS and inhibit release of pro-inflammatory mediators from human and murine macrophages.

## Results

### Immuno-stimulatory activity of meningococcal capsular polysaccharides is neutralized by LL-37

**Figure 1:**
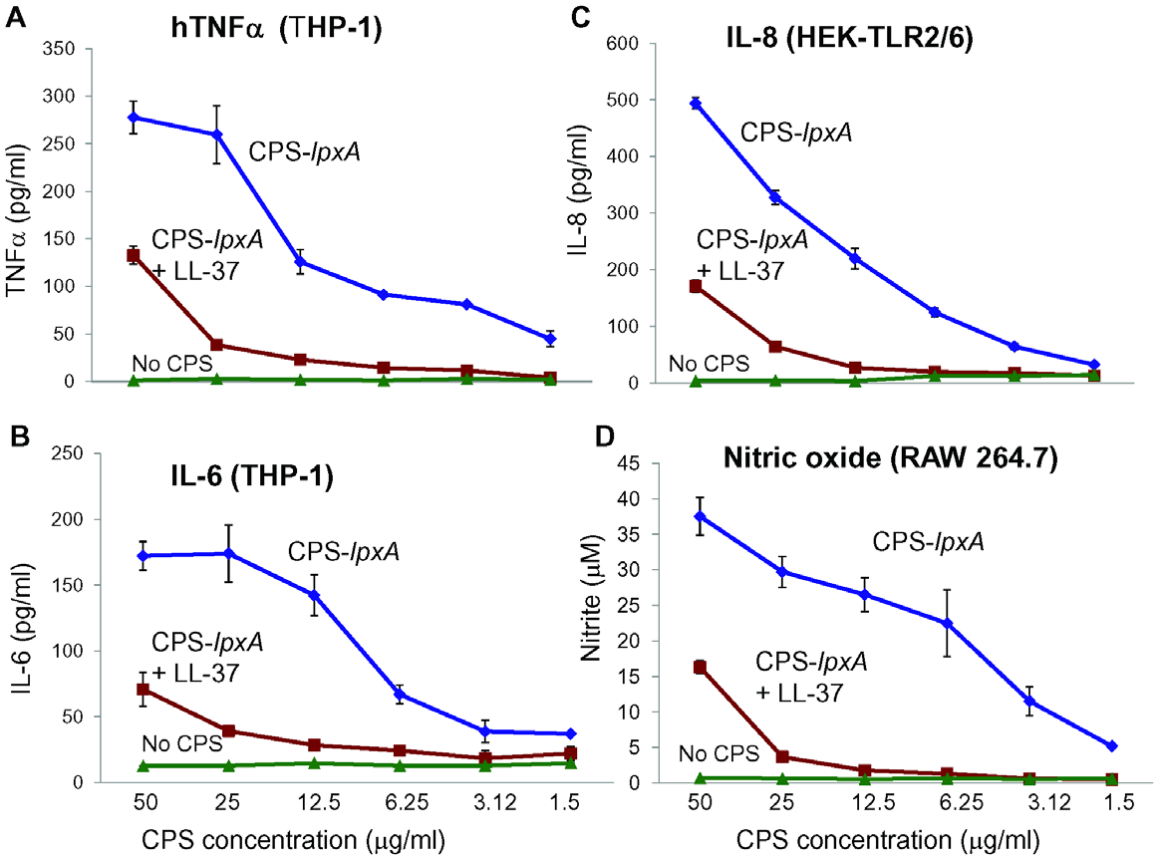
LL-37 neutralized meningococcal CPS bioactivity and inhibited cytokine release from human and murine macrophages. CPS polymers were purified from the endotoxin-deficient serogroup B meningococcal NMB-*lpxA* mutant designated CPS-*lpxA*. **A**: TNFα release from human macrophage-like THP-1 cells induced overnight with CPS-*lpxA* polymers pre-incubated with or without 2 µg/ml of LL-37 for 30 min at 37°C (Materials and Methods section). **B**. IL-6 release from THP-1 cells induced as in panel A. **C**. IL-8 release from HEK-TLR2/6 stably transfected cells induced with CPS-*lpxA* as in panel A. TNFα,IL-6 and IL-8 release was measured by ELISA. **D**. Nitric oxide release from murine RAW 264.7 macrophages induced with CPS-*lpxA* polymers as in panel A and measured by the Griess method as nitrite accumulation after 24 h of incubation at 37°C with 5% CO_2_. Error bars represent ±SD from the mean of quadruplicate measurements. This experiment is representative of three independent experiments.

Meningococcal CPS polymers consist of distinct repeating glucan units that vary in composition and linkage among invasive serotypes [1]. Meningococcal CPS polymers are polyanionic due to the negatively charged sialic acid residues found in many invasive meningococcal serogroups (e.g. B, C, Y, W-135) or due to the presence of phosphates in the serogroup A polymers [1], [4], [12]. The immuno-stimulatory activity (bioactivity) of meningococcal CPS polymers (vaccine grade and laboratory prepared CPS) was investigated *in vitro* using well established and characterized human and murine cell lines [14]. CPS was purified from an endotoxin-deficient serogroup B *N. meningitidis lpxA* mutant (designated CPS-*lpxA*) and consists of (α2→8)-*N*-acetylneuraminic acid rendered protein-, lipopeptide-, phospholipid- and nucleic acid-free by digestion with proteinase K, DNAse and RNAse during the extraction and purification procedures (see Methods). This CPS induced a dose-dependent release of the pro-inflammatory cytokines TNFα and IL-6 from THP-1, human macrophages-like cells (Figure 1A and B), IL-8 from HEK-TLR2/6 stably transfected cells (Figure 1C) and nitric oxide (NO) release from murine RAW 264 macrophages (Figure 1D). Hence, based on its ability to induce macrophage activation, as assessed by release of cytokines and NO, we concluded that the endotoxin-free serogroup B CPS is bioactive in an LPS-independent manner; other CPS polymers from serogroup A, C, Y and W-135 strains were found to have a similar activity (see below).

**Figure 2:**
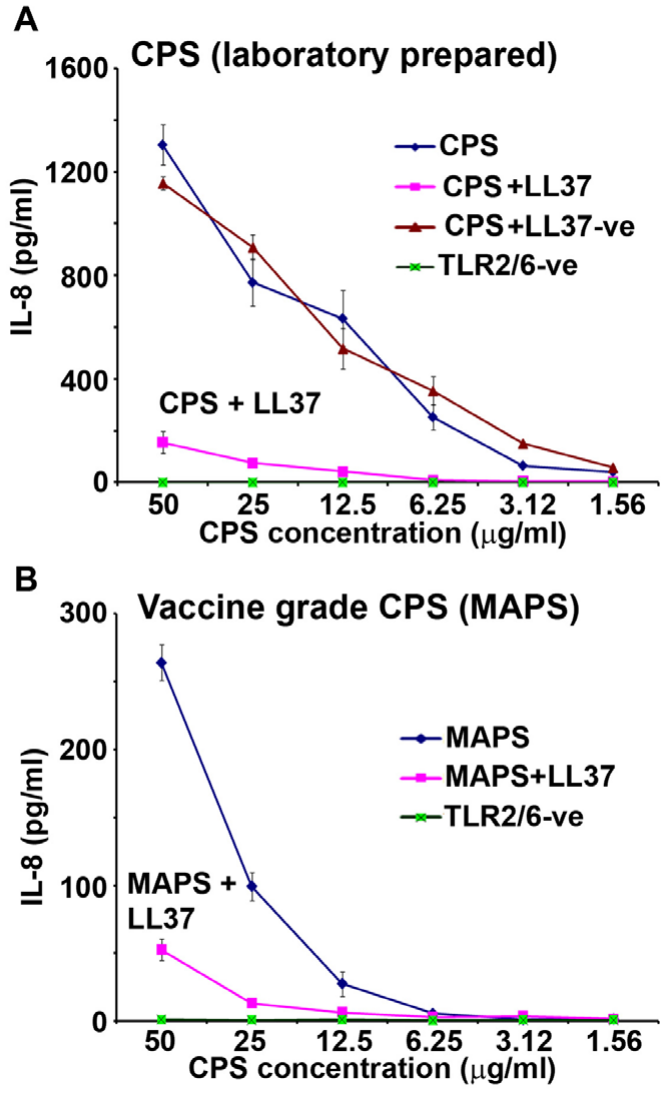
Cationic charge is important for LL-37 interaction with CPS polymers. **A:** IL-8 release from HEK-TLR2/6 stably transfected cells induced with serogroup A meningococcal CPS polymers pre-incubated with 4 µg/ml of either LL-37 parent analog or the negatively charged LL-37R/D-K/E (LL-37-ve) analog used as a control. **B:** IL-8 release from HEK/TLR2/6 cells induced with a vaccine grade CPS purified from *N. meningitidis* serogroup A (MAPS) pre-incubated with or without 4 µg/ml of synthetic LL-37 parent analog. TLR2/6-ve is unstimulated HEK-TLR2/6 stably transfected cells. IL-8 measured by ELISA. Error bars represent ±SD from the mean of quadruplicate measurements. This experiment is representative of two independent experiments.

Pre-incubation of the serogroup B meningococcal CPS-*lpxA* polymers with the human cathelicidin LL-37 resulted in dramatic attenuation of CPS bioactivity. Thus, pre-incubation of CPS-*lpxA* with 2 µg/ml of synthetic LL-37 inhibited cytokines and nitric oxide release from target cells in a dose-dependent manner (Figure 1A–D). The positively charged LL-37 peptide, but not the negatively charged analog LL-37R/D-K/E, neutralized the capacity of CPS in inducing the release of pro-inflammatory chemokine IL-8 (Figure 2A and Figure S3). Thus, the positive charge of LL-37 was important to the ability to neutralize meningococcal CPS polymers.

Importantly, LL-37 and certain analogs neutralized the immuno-stimulatory activity of meningococcal CPS polymers without evidence of cellular toxicity as assessed by Trypan Blue exclusion and LDH release assays (13 and data not shown). Vaccine grade CPS purified from a serogroup A strain consisting of (α1→6)-*N*-acetyl-D-mannosamine-1-phosphate (designated MAPS) was also used in our studies. IL-8 release from HEK/TLR2/6 cells induced by MAPS was significantly reduced when the CPS (MAPS) was pre-incubated with 2 µg/ml of LL-37 (Figure 2B). The results suggest that meningococcal CPS charge rather than glucan structure or repeat unit of the CPS was important for its interaction with LL-37. This hypothesis was tested in subsequent experiments (see below).

### The antibacterial domain of LL-37 is required for interaction with meningococcal CPS polymers

**Figure 3:**
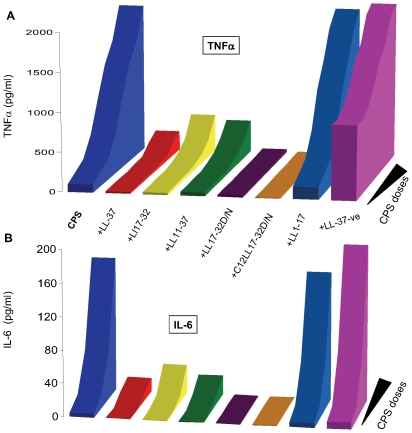
LL-37 active domain is required for interaction with meningococcal CPS polymers. Acylation and charge of truncated LL-37 analogs enhanced interaction with meningococcal CPS polymers and inhibited TNFα(**A**) and IL-6 (**B**) release in a dose-dependent manner. THP-1 cells stimulated with meningococcal serogroup B, endotoxin-free CPS-*lpxA* doses ranging from 100 µg to 50 ng/ml with or without 10 µg/ml synthetic LL-37 analogs. LL-37-ve is negatively charged analog LL-37 K/E-R/D used as a control. TNFα and IL-6 release was quantified by ELISA. Error bars represent ±SD from the mean of quadruplicate readouts. The results are representative of 4 independent experiments.

**Figure 4:**
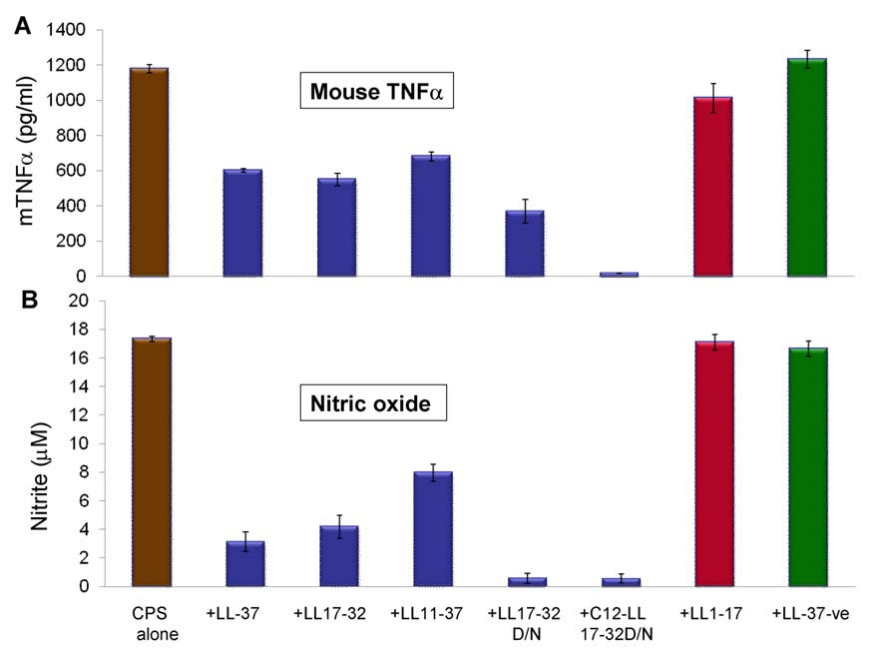
LL-37 active domain is required for interaction with meningococcal CPS polymer and inhibited immune responses in murine macrophages. A: Mouse TNFα release from murine RAW 264 macrophages induced with 20 µg /ml serogroup B meningococcal CPS-*lpxA* polymers pre-incubated with 2 µg/ml synthetic LL-37 analogs for 30 min at 37°C prior to cellular induction. B: Nitric oxide release from stimulated RAW 264.7 used in panel A was quantified by the Greiss method. Error bars represent the ±SD from the mean of 4 independent wells. The results are representative of 2 independent experiments.

**Figure 5:**
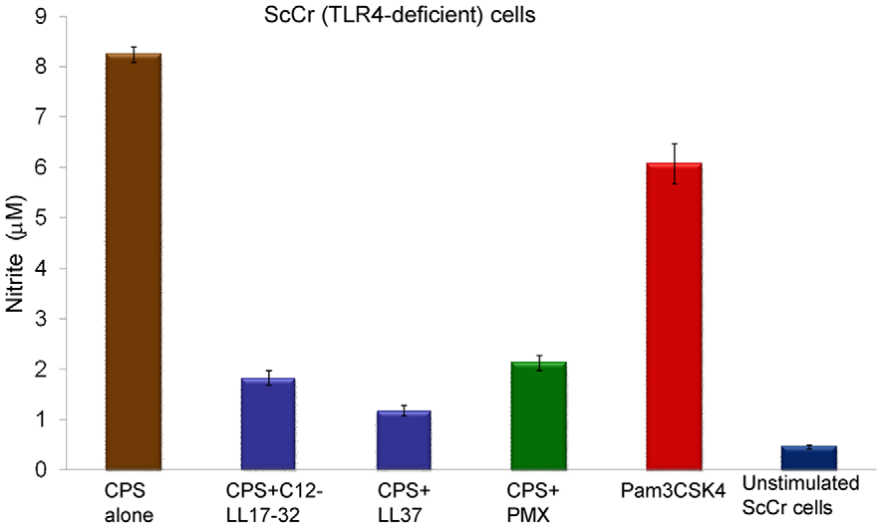
LL-37 interaction with meningococcal CPS polymer is TLR4-independent. Nitric oxide release from murine ScCr (TLR4-deficient) cells induced with with 20 µg /ml serogroup B meningococcal CPS-*lpxA* polymers pre-incubated with 2 µg/ml synthetic LL-37 analogs for 30 min at 37°C prior to cellular induction. PMX: polymyxin B; Pam3CSK4: synthetic lipopeptide and a TLR2 ligand used as a control at 2 µg/ml final concentration. Error bars represent the ±SD from the mean of 4 independent wells. The results are representative of 2 independent experiments.

The ability of synthetic LL-37 analogs (Table 1) to interact with meningococcal CPS polymers and neutralize bioactivity or inhibit cytokines and nitric oxide release was investigated. The antibacterial domain of LL-37 was previously described [15] and consists of amino acid residues 17–32, which was confirmed in the present study using *N. gonorrhoeae* as a target pathogen (Table S1). In a dose-dependent manner, truncated LL-37 analogs that contain the active domain amino acid sequences 17–32, but not residues 1–17 (inactive domain), neutralized endotoxin-free CPS immuno-stimulatory activity as assessed by release of pro-inflammatory mediators TNFα (Figure 3A) and IL-6 by target cells (Figure 3B). Further, acylation with a highly nonpolar C12 alkyl moiety of a truncated analog (C12LL17-32), as well as replacement of the negatively charged residue, aspartic acid (D), with the polar, non-charged residue, asparagine (N), C12LL17-32D/N analog, enhanced interaction with meningococcal CPS polymers as indicated by their ability to inhibit release of TNFα (Figure 3A) and IL-6 (Figure 3B) from human THP-1 cells. The ability of truncated LL-37 analogs to neutralize meningococcal CPS polymers correlated with their antibacterial action (Table S1) against *N. gonorrhoeae*, which naturally lacks CPS [16]. Taken together, the data suggest that both charge and amphipathicity are important for LL-37 analogs antibacterial activity and interaction with meningococcal CPS polymers. Similarly, synthetic truncated LL17-32 analogs inhibited CPS induced TNFα release (Figure 4A) and nitric oxide release (Figure 4B) from murine RAW 264, a TLR4-suficient macrophage cell line. An acylated, truncated analog of LL-37 (C12LL17-32) also inhibited CPS-induced nitric oxide release from murine ScCr, a TLR4-deficient macrophage cell line (Figure 5).

**Table 1:** Synthetic LL-37 variants amino acid sequences.

LL-37	L^1^LGDFFRKSKEKIGKEFKRIVQRIKDFLRNLVPRTES^37^
LL-37R/D-K/E (-ve)	L^1^LGDFFDESEEEIGEEFEDIVQDIEDFLDNLVPDTES^37^
LL-37 R/D	L^1^LGDFFDKSKEKIGKEFKDIVQDIKDFLDNLVPDTES^37^
LL-37 K/E	L^1^LGDFFRESEEEIGEE FERIVQRIEDFLRNLVPRTES^37^
LL-37 K/A	L^1^LGDFFRASAEAIGAE FARIVQRIADFLRNLVPRTES^37^
LL-37 K	L^1^LGKFFKKSKKKIGKKFKKIVQKIKKFLKNLVPKTKS^37^
LL-37 R	L^1^LGRFFRRSRRRIGRRFRRIVQRIRRFLRNLVPRTRS^37^
LL 17-32	F^17^KRIVQRIKDFLRNLV^32^
LL 17-32D/N	F^17^KRIVQRIKNFLRNLV^32^
C12LL 17-32D/N	dodecanoyl-F^17^KRIVQRIKNFLRNLV^32^
LL 11-37	E^11^KIGKE FKRIVQRIKDFLRNLVPRTES^37^
LL 1-17	L^1^LGDFFRKSKEKIGKEF^17^

### Synthetic LL-37 analogs interaction with vaccine grade meningococcal CPS

We hypothesized that because LL-37 can be produced by professional immune cells, the ability of CPS-based bacterial vaccines (conjugated and non-conjugated) to modulate innate immunity responses might be influenced by LL-37. To test this hypothesis, the interaction between meningococcal CPS polymers and LL-37 and the truncated analogs (Table 1) was examined. For this purpose, we used the MAPS CPS purified from serogroup A *N. meningitidis* and compared it to laboratory-prepared CPS from a serogroup A strain (NMA). Truncated LL-37 analogs that contain the antibacterial domain (residues 17–32) inhibited both the laboratory prepared NMA CPS and the vaccine grade meningococcal CPS (MAPS) and reduced nitric oxide release from murine RAW 264 macrophages (Figure S1A and S1B). Similar reductions in TNFα (Figure S1C) and IL-6 (data not shown) release from THP-1 cells were seen. Synthetic LL-37 variants inhibited commercial MAPS CPS similar to the laboratory purified meningococcal CPS polymers. Cationic peptides such as synthetic LL-37 variants and polymyxin B inhibited CPS polymers, but this was not seen with ampicillin, a classical zwitterion (Figure S1B).

The cationic nature of LL-37 was anticipated to be a critical determinant in the ability of LL-37 to interact with CPS and neutralize its bioactivity. To test this, a synthetic LL-37 analog (LL-37K/E), where all positively charged lysine (K) residues were replaced with the negatively charged acidic residue glutamic acid (E), was examined for its ability to inhibit the pro-inflammatory capacity of CPS. This peptide showed reduced neutralizing activity against purified CPS polymers. The reduced ability of LL-37K/E to interact with purified CPS correlated with reduced bactericidal activity against gonococcal strain FA19 (Table S1) where the LL-37 MIC is  = 7.8 µg/ml and the LL-37K/E MIC is >1000 µg/ml. In contrast, the replacement of all cationic and anionic residues by lysine (LL-37K) or arginine (LL-37R) did not enhance the neutralizing activity of the analog against purified CPS polymers, but retained excellent MIC activity against gonococci (Table S1).

### LL-37 analogs interact with CPS from various meningococcal invasive serogroups

**Figure 6:**
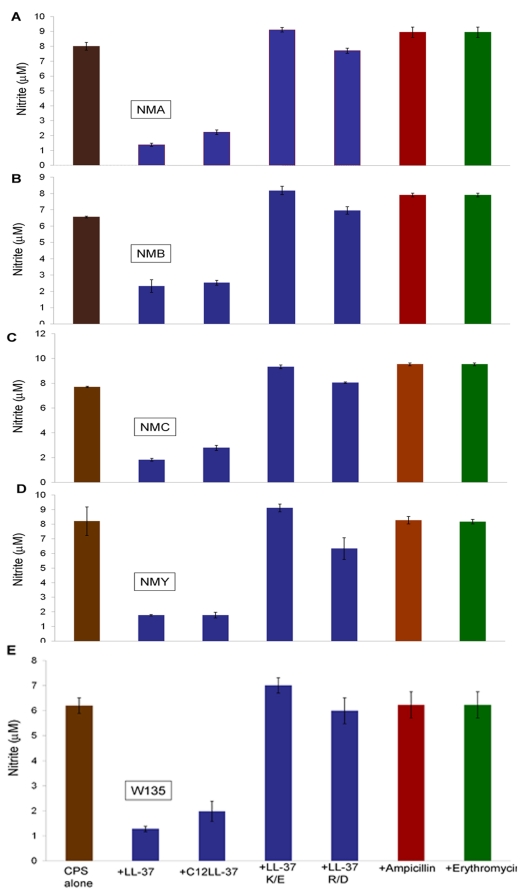
Meningococcal CPS serogroups A, B, C, Y and W135 interact with LL-37 analogs. Laboratory prepared meningococcal CPS polymers (20 µg/ml) from invasive meningococcal serogroups were pre-incubated with 4 µg/ml of LL-37 analogs and used to stimulate ScCr (TLR4-deficient) murine macrophages overnight. **A-** NMA CPS is meningococcal serogroup A CPS; **B-** NMB CPS is meningococcal serogroup B; **C-** NMC CPS is meningococcal serogroup C CPS; **D-** NMY CPS is meningococcal serogroup Y CPS and **E-** W135 CPS meningococcal serogroup W135 CPS. Nitric oxide release was measured as nitrite accumulation in the supernatants using the Greiss method. A non-cationic antibiotics ampicillin and erythromycin were used at 20 µg/ml as controls. Error bars represent the ±SD from the mean of 4 independent wells. This is representative of three independent experiments.

Since the meningococcal capsule is the basis for serogroup designation, we tested whether interaction of LL-37 analogs with CPS was dependent on capsule structure. Laboratory prepared CPS polymers from invasive meningococcal strains A, B, C, Y and W135 were tested in the TLR4-deficient macrophage cell line ScCr to eliminate the contribution from any LPS contamination in the preparations. Twenty micrograms of the CPS polymers were pre-incubated with 4 µg/ml of LL-37 analogs for 30 min, then used to stimulate ScCr cell overnight; as controls, CPS polymers were also pre-incubated with 20 µg/ml of ampicillin or erythromycin. Truncated and acylated LL-37 analogs significantly reduced nitric oxide release induced by various meningococcal CPS (Figure 6); this inhibition was not observed with ampicillin and erythromycin. Similar results were seen in epithelial HEK/TLR2/6 transfected cells stimulated with different meningococcal CPS structures. Pre-incubation with LL-37 resulted in a significant reduction in IL-8 release. Taken together, the data indicate that LL-37 interaction with meningococcal CPS polymers is independent of the repeating carbohydrate unit structure in CPS, but is more likely a reflection of the negative charge of meningococcal CPS polymers.

### Synthetic LL-37 analogs decrease NFκB activation induced with meningococcal CPS

**Figure 7:**
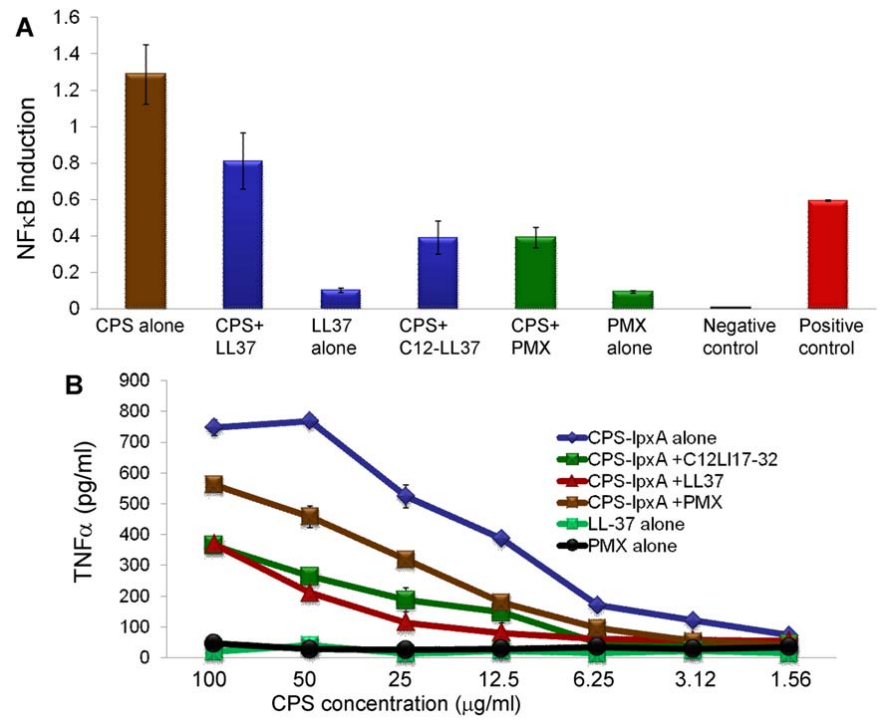
Synthetic LL-37 inhibited NFκB activation and TNFα release by meningococcal CPS-*lpxA* polymers. **A:** Inducible NFκB dual luciferase reporter transfected into HEK-TLR2/6 stably transfected cells then stimulated with serogroup B meningococcal CPS-*lpxA* with or without LL-37 analogs. CPS-*lpxA* polymer (100 µg) was pre-incubated with 10 µg of LL-37, C12-LL17-32 or polymyxin B (PMX) prior to cellular induction for 5 h. Inducible NFκB firefly luciferase activity was normalized to constitutively expressing *Renilla* luciferase reporter. Negative control is transfected with non-inducible firefly luciferase reporter. Positive control is a mixture of constitutively expressing GFP and firefly luciferase construct. **B:** TNFα release from human THP-1 cells induced with CPS-*lpxA*-LL-37 analog prepared complexes used in panel A. TNFα protein was quantified by ELISA and error bars represent ±SD from the mean of 4 independent wells. This experiment is representative of three independent determinations.

The neutralizing activity of LL-37 analogs against meningococcal CPS was confirmed in human epithelial HEK/TLR2/6 cells transfected with the inducible NFκB dual luciferase reporter. Pre-incubation of meningococcal endotoxin free CPS polymers (20 µg/ml) with synthetic LL-37 analogs (4 µg/ml) for 30 min prior to cellular stimulation resulted in attenuation of the NFκB inducible luciferase reporter (Figure 7A). The truncated LL-37 analogs that contain the active domain LL-17-32 as well as the acylated C12LL17-32 molecule and polymyxin B (PMX) were able to inhibit meningococcal CPS induced NFκB induction and decreased TNFα release (Figure 7B).

### Meningococcal CPS polymers bind to LL-37

**Figure 8:**
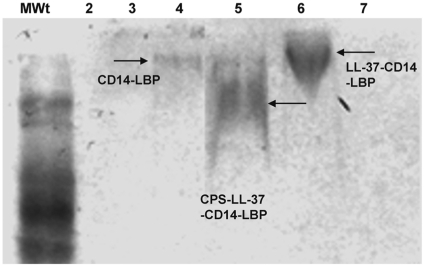
Electrophoretic mobility gel shift assay of meningococcal CPS-*lpxA* polymers in the presence of LL-37, LBP and CD14. CPS-*lpxA* complexes were prepared as described in the Methods section, were run on 8% native PAGE, and detected by Western blot using biotinylated LBP antibody. Lane **1** (MWt): molecular weight marker; lane **2** CPS-*lpxA* alone; lane **3**: CPS-*lpxA* **-**CD14-LBP; lane **4**: CD14-LBP alone; lane **5**: CPS-*lpxA* **-**LL-37-CD14-LBP; lane **6**: LL-37-CD14-LBP; lane **7**: CPS-*lpxA* **-**LL-37 alone.

LL-37 and other cationic peptides have the ability to bind LPS by virtue of their cationic and hydrophobic properties [17]. To test if LL-37 and CPS can form a complex, we employed an electrophoretic mobility shift assay (EMSA) to resolve potential CPS:LL-37 complexes. Since CPS polymers are hydrophilic and highly water soluble, CD14 and LBP were used to stabilize the carbohydrate polymers for the EMSA assay [18]. Complexes were separated by 8% native PAGE and Western blotting using biotinylated anti-LBP and anti-CD14 antibodies to detect the complexes. Both CD14 and LBP were required to stabilize CPS complexes and visualize the shift. The additions of LL-37 to CPS-*lpxA*-CD14-LBP complexes caused a gel-shift (Figure 8, lane 5) compared to CPS-*lpxA*-CD14-LBP complexes (Figure 8 lane 3), to CD14-LBP alone (Figure 8, lane 4) and to LL-37-CD14-LBP complexes (Figure 8, lane 6). The bioactivity of the CPS-*lpxA* complexes used in gel-shift was further confirmed using RAW 264 macrophages. In contrast to CPS-*lpxA* complexes alone or with CD14 and LBP that were very active and induced large nitric oxide release, CPS-*lpxA* complexes that contained LL-37 gel-shifted in lane 5, showed significant reduction in nitric oxide release (data not shown). The results demonstrate that LL-37 binds to meningococcal CPS-*lpxA* polymers in the absence of endotoxin and that this specific interaction results in decreased CPS immuno-stimulatory activity. Thus, the interaction of this host-derived peptide with meningococcal CPS modulates innate immune responses.

## Discussion

Bacterial capsular polysaccharides (CPS) have been extensively studied as virulence factors due to their ability to impede organism clearance by host antimicrobial systems, such as phagocytosis by professional phagocytes and complement mediated killing. Bacterial CPS also form the basis for vaccines including those that protect against serogroups A, C, Y and W-135 meningococci [1]. Increasing evidence also suggests that CPS functions as an immune modulator with pro-inflammatory activities similar to other bacterial products (e.g., endotoxin). Indeed, results presented herein demonstrate that laboratory-prepared meningococcal CPS devoid of LPS can activate human and murine macrophages as assessed by release of cytokines and NO through TLR2- and TLR4-MD2 dependent pathways. This activation of pro-inflammatory signaling pathways can result in release of immune modulators (e.g., cytokines, chemokines, NO and ROS) that can impact severity of disease (e.g., sepsis and meningitis). Also, there is increasing evidence that cationic antimicrobial peptides that are constitutively produced by phagocytes or inducibly synthesized by epithelial cells can dampen the ability of bacterial ligands (e.g., endotoxin and LTA) to amplify innate immune responses [7], [17]. To our knowledge, this is the first report of a host defense peptide (LL-37) that has the ability to dampen the immune-stimulatory activity of bacterial CPS.

Extensive efforts were made to eliminate other TLR ligands in all CPS preparations which might have confounded the results. These efforts included digestion of samples with proteinase K, DNAse and RNAse treatments, ultracentrifugation, gel filtration, and extensive dialysis that are expected to remove low-MW compounds and retain large CPS polymers. The purity of CPS preparations was also demonstrated by Alcian blue and silver staining [19] of PAGE gels containing CPS preparation (data not presented). Further, CPS extraction from the LOS-deficient meningococci was performed in parallel to a phospholipid extraction (designated PL-*lpxA*) from the same GC broth. In contrast to CPS-*lpxA*, PL-*lpxA* induced a TLR2-mediated response, but LL-37 failed to neutralize its biologic activity. Thus, TLR ligand contamination in these preparations was ruled out.

Using synthetic analogs of the amphipathic, alpha-helical LL-37 peptide, we observed that the cationic and hydrophobic properties are important characteristics in the ability to detoxify or neutralize meningococcal CPS polymers. The antibacterial domain (defined by residues 17–32) of LL-37 was required for detoxification of CPS. Importantly, LL-37 and its truncated analogs did not impact the viability of human or murine macrophage at concentrations that were effective in detoxifying CPS. The capacity of LL-37 to detoxify CPS required electrostatic and hydrophobic interactions. Indeed, LL-37 analogs that differed in their cationic and/or hydrophobic properties differed in their ability to neutralize CPS. Truncated and acylated LL-37 analogs neutralized meningococcal CPS immuno-stimulatory activity and inhibited cytokine release and inflammatory mediators by inhibiting NFκB activation. Thus, no selective inhibition or induction of signaling pathways like the MyD88 or TRIF-dependent pathway was observed. Pre-treatment of macrophages with LL-37 for 30 min followed by washing to remove LL-37 prior to stimulation with meningococcal CPS polymers did not affect cytokine release (Figure S2). Thus, the complexes formed between LL-37 and meningococcal CPS polymers lead to the neutralization of immuno-stimulatory activity and decreased cytokine release.

In support of our findings and conclusions, Foschiatti et al. [20] recently used circular dichroism, fluorescence spectroscopy and atomic force microscopy to demonstrate the direct binding of LL-37 to exopolysaccharides of *Pseudomonas aeruginosa* and *Burkholderia cepacia* complex. The negative charges of exopolysaccharides were found to be important for complex formation with LL-37 and the binding was through electrostatic and other non-covalent interactions [20]. The impact of LL-37 binding to polysaccharides was also demonstrated in an animal model of *Pseudomonas* sinusitis biofilm, where a high concentration of LL-37 derived peptide was shown to eradicate *Pseudomonas* biofilms and decrease bacterial viability [21]. Using an EMSA procedure, we confirmed the ability of meningococcal CPS polymers to directly bind LL-37. Meningococcal CPS are negatively charged polymers and meningococci are highly resistant to AMPs [8]. Host cationic peptides contribute to bacterial killing directly by destabilizing bacterial membranes [7] or by forming phagocyte extracellular traps (ETs). These traps are formed when AMPs bind to host DNA, histones and proteases to form a matrix that entraps and kills invading pathogens [22]. Thus, LL-37 binding to anionic CPS polymers may deplete the availability of these peptides to form ETs or even to penetrate and reach their bacterial membrane target.

In addition to their direct antimicrobial action, host cationic peptides such as LL-37 can also modulate various immune responses and in some cases lead to pathological consequences. For example, Lande et al. reported that LL-37 coupled with self-DNA released from necrotic host cells induced plasmacytoid dendritic cells that drove autoimmunity in psoriasis [23]. In a more recent study the same authors found that self-RNA also complex with LL-37 and induce TLR7 and TLR8 [24]. DNA and RNA molecules are highly negatively charged (polyanionic) and thus would electrostatically bind to LL-37. Sandgren et al. demonstrated that LL-37 facilitates transfer of extracellular DNA plasmid to the nuclear compartment of mammalian cells via lipid rafts and endocytosis [25]. Further, LL-37 has been shown to complex with polyanionic DNA and F-actin bundles accumulated in cystic fibrosis airway fluid, thus leading to inactivation of these cationic peptides [26], [27]. In further support, Hurtado et al. reported the rapid interaction of LL-37 with bacterial DNA unmethylated CpG oligonucleotides motifs that contain the negatively charged phosphodiesters and phosphorothionate backbones. They concluded that LL-37 aids the delivery of CpG motifs to the endosomal compartment and rapidly induced TLR9 in B lymphocytes and plasmacytoid dendritic cells [28]. Other recent studies have documented a role for host-derived cationic peptides in gut homeostasis [29], [30]. Microbiota, although symbiotically living in the bowel, continuously shed capsular polysaccharides. The shed capsular polymers are neutralized and degraded by an unknown mechanism. Thus, it is possible that cationic peptides released from the gut mucosal surfaces to the lumen bind to CPS and play a key role in dampening inflammation and maintaining homeostasis, hence the physiological role of LL-37 in keeping gut inflammation at bay [31], [32]. Taken together, LL-37 can form complexes with various pathogen-derived anionic polymers leading to immune response modulation.

In summary, LL-37 and certain analogs modulate the capacity of meningococcal CPS polymers to induce innate immune responses. Based on our observations, this host defense peptide could be considered in the future as a therapeutic option for dampening the pathologic consequences of inflammation and sepsis.

## Materials and Methods

### Reagents

RPMI 1640 medium, Dulbecco's Eagle medium, fetal bovine serum (FBS), penicillin/streptomycin, sodium pyruvate and nonessential amino acids were obtained from Cellgro Mediatech (Herndon, VA). Polymyxin B, ampicillin, and erythromycin were purchased from Sigma Chemical (St. Louis, MO). Phorbol myristate acetate (PMA) was purchased from GibcoBRL (Grand Island, NY). Human and mouse TNFα IL-8, IL6 and IP-10 ELISA kits were from R&D Systems (Minneapolis, MN). THP-1, RAW 264.7, and 23ScCr (TLR4-deficient) cell lines were purchased from ATCC (Mannasas, VA). Basticidin, human embryonic kidney 293 HEK-TLR2/6 and HEK-TLR2 stably transfected cells were purchased from InvivoGen (San Diego, CA). HEK-TLR2/CD14 stably transfected cell line was provided by Dr. Evelyn Kurt-Jones (University of Massachusetts Medical Center, Worcester, MA).

### Cationic peptides

The solid-phase peptide synthesis of LL-37 and its analogs (Table 1), used in this study, has been previously described [17]. The peptides were purified to >98% chromatographic homogeneity by reversed-phase HPLC on C18-silica packings and were obtained in the form of their trifluoroacetate salts; their masses were confirmed by MALDI-TOF mass spectrometry. The peptides were dissolved in 0.01 % (v/v) acetic acid and stored at −20°C prior to use. Antibacterial assays were performed as described previously [33] and used *N. gonorrhoeae* strain FA 19. All assays were performed in triplicate and values are reported as the minimal inhibitory concentration (MIC).

### Capsular polysaccharide purification

CPS was purified from an endotoxin-deficient serogroup B *Neisseria meningitidis* mutant (NMB-*lpxA*) and from other meningococcal serogroups like A, B, C, W135 and Y as previously described [34]. Briefly, bacteria were grown in 3 liters of GC broth for 16 hours and CPS was released by lysing with 10% of Cetavlon (hexadecyl-trimethyl ammonium bromide) added to a final concentration of 1.0%. The precipitate and bacterial debris were collected by centrifugation (11,000 × *g* for 15 min) and then resuspended in 50 ml of distilled water. CPS-Cetavlon complexes were dissociated with 1 volume of 2 M CaCl_2_ and stirring for 1 h. Nucleic acids were precipitated with absolute ethanol and removed by centrifugation. CPS were precipitated by 80% ethanol, washed 3 times with acetone and twice with diethyl ether and dried by vacuum. Contaminating proteins and phospholipids were removed and the purified CPS was further subjected to proteinase K digestion, DNAse and RNAse treatment followed by extensive dialysis [19]. Vaccine grade CPS (MAPS) was a gift from Dr. Seshu Gudlavalleti, the Center for Biologics, FDA (now at GN International Medical Corp. Omaha, NE).

### Cell cultures

THP-1, human macrophage-like cells were grown in RPMI 1640 with L-glutamate supplemented with 10% FBS, 50 IU/ml of penicillin, 50 µg/ml of streptomycin, 1% sodium pyruvate and 1% non-essential amino acids. Culture flasks were incubated at 37°C with humidity under 5% CO_2_. Murine macrophages (RAW 264.7 and 23ScCr) and human kidney epithelial cells HEK293 were grown in Dulbecco's Eagle medium supplemented and incubated as noted above.

### Cellular activation

Human THP-1 (macrophage-like cells) and murine RAW 264.7 (TLR4-sufficient), 23ScCr (TLR4-deficient) and HEK-TLR2/6/CD14 stably transfected cell lines were stimulated with meningococcal CPS polymers with or without pre-incubation with LL-37 and its analogs (Table 1). CPS polymers were purified from the LPS-deficient serogroup B *Neisseria meningitidis lpxA* mutant as well as other meningococcal serogroups A, B, C, W135 and Y. Purified CPS samples were freshly dissolved in pyrogen-free sterile H_2_O at 1 mg/ml stock concentration and vortexed for 2 min. Working CPS concentrations (ranging from 100 µg/ml to 50 ng/ml) were made in duplicate wells using sterile PBS by serial fold dilutions in the 96-well tissue culture plates (Becton Dickinson, Franklin Lakes, NJ) at 50 µl final volumes. Cationic peptides (2, 4 or 8 µg/ml) or PBS equivalent volumes were added to designated wells and pre-incubated for 30 min at 37°C. Freshly grown THP-1 cells and HEK-TLR2/6 transfected cells as well as murine macrophages, each adjusted to 10^6^ cell/ml and 250 µl aliquots were dispensed into each well at final 250×10^3^ cell density in the designated 96-well plates. The plates were incubated overnight at 37°C with 5% CO_2_ and humidity. Supernatants from stimulated cells were harvested and stored at −20°C until use.

### Cytokine profiles

The cytokines TNFα, IL-6 and CXCL10 (IP-10), released from THP-1 cells, and IL-8, released from HEK-TLR2 transfected cells stimulated with meningococcal CPS polymers, were quantified by DuoSet ELISA (R&D Systems) as previously described [14].

### Nitric oxide induction by murine macrophages

Freshly grown adherent RAW 246.7 or 23ScCr (TLR4-deficient) macrophages were harvested, washed and re-suspended in Dulbecco's complete media, counted and adjusted to 10^6^ cell/ml. 250 µl aliquots were then dispensed into each well of a 96-well plate at final 250×10^3^ cell density prior to stimulation with purified CPS polymers with or without cationic peptides. The induced RAW 264.7 or 23ScCr macrophages were incubated overnight at 37°C with 5% CO_2_ and supernatants were harvested and saved. Nitric oxide release was quantified using the Greiss chemical method as previously described [9].

### Cell-based multi pathway activity assay

To determine the signaling pathways induced upon meningococcal CPS recognition, a transcription factors array (Cignal Finder™ 10-Pathway Reporter Arrays, SABiosciences, Fredrick, MD) consisting of 10 dual-luciferase reporters assays were used according to the manufacturer instructions. Each of the 10 reporter assays encodes for an inducible transcription factor responsive firefly luciferase reporter and constitutively expressing *Renilla* construct in 40∶1 ratio. Briefly, DNA reporter master mixes were prepared in SureFect transfection reagent (SABiosciences) diluted in Opti-MEM media without serum or antibiotics and 25 µl/well were dispensed into 96-well white tissue culture plates. For reverse transfection, freshly grown cells were counted and adjusted to 1×10^6^ cells/ml in Opti-MEM without serum or antibiotics. 100 µl of cells (100×10^3^ cells/well) were laid over the DNA reporters in 96-well plates and incubated overnight at 37°C with 5% CO_2_. Transfection media were removed and replaced with 150 µl of fresh D-MEM supplemented with 10% FBS and incubated again overnight at 37°C. Cells were then induced with meningococcal CPS polymers or LOS doses for 5 h and dual-luciferase reporter activity was determined using Dual-Luciferase reporter assay system (Promega Corporation, Madison, WI) following the manufacturer instructions. Induced transcription factors were reported as firefly luciferase activity normalized to *Renilla* luciferase activity.

### Electrophoretic mobility shift assay (EMSA) of CPS complex with LL-37, CD14 and LBP

To assess binding of CPS to LL-37 in the presence of human CD14 and LBP, an EMSA assay using native gel conditions was performed [18]. Serogroup B CPS purified from a LOS-deficient meningococcal *lpxA* strain were dissolved in sterile H_2_O at 1 mg/ml concentration and 20 µl were transferred into an Eppendorf tube mixed with 10 µl (5 µg) of LL-37, 10 µl (0.5 µg) of human rCD14 and 10 µl (0.5 µg) of rLBP (R&D Systems) in 30 µl final volume and incubated for 3 h at 37°C. An aliquot of 7.5 µl of the mixed complexes were transferred into a new Eppendorf tube and mixed with an equal volume of sample buffer (0.1 M Tris-glycine buffer containing 20% glycerol and bromophenol blue) and 15 µl of the mixture were loaded on 8% native polyacrylamide gel electrophoresis (PAGE) without SDS as previously described [18]. Electrophoresis was performed with Tris-glycine buffer pH 8.8 without SDS and run for 2 h at 4°C. The gel-shifting of CPS complexes was detected by Western blot method using biotinylated anti CD14 and anti LBP antibodies (R&D Systems) diluted 1∶1500× with blocking buffer.

### Statistical analysis

Mean values ± SD and *P* values (Student *t* test) of at least four independent determinations were calculated with Microsoft Excel software.

## Supporting Information

Figure S1 Synthetic LL-37 analogs interact with a vaccine grade meningococcal CPS. Murine RAW 264 macrophages stimulated with (A) laboratory prepared serogroup A meningococcal CPS polymers or (B) a vaccine grade serogroup A meningococcal CPS 20 μg /ml (MAPS) pre-incubated with or without 4 μg/ml of synthetic LL-37 analogs. Ampicillin, a non-cationic antibiotic, was used as a control. Nitric oxide release from induced murine cells was quantified by the Griess method as nitrite accumulation after 24 h of incubation at 37°C with 5% CO2. RAW-ve is unstimulated cells but 50 μl of equivalent PBS volume is added. C: TNFα release from human THP-1 cells stimulated with laboratory prepared meningococcal serogroup A CPS polymers pre-incubated with LL-37 analogs as in panel A. Error bars represent the ±SD from the mean of 4 independent wells. The results are representative of three independent experiments. * p values were calculated using Excel software student t-test in reference to CPS alone without LL-37 analogs.
(6.85 MB TIF)

Figure S2 Pre-treatment of macrophages with LL-37 did not alter cellular responses. Murine macrophages RAW 264 were pre-treated with 10 μg/ml of LL-37 or its inactive analog (the negatively charged LL-37-ve) for 30 min, followed by washing to remove LL-37 prior to stimulation with meningococcal LOS doses (A) or CPS-*lpxA* dose of 50 μg/ml (B). Nitric oxide release was measured as nitrite accumulation in supernatants and quantified by the Greiss method.
(0.60 MB TIF)

Figure S3 LL-37 interacts with meningococcal CPS and inhibits IL-8 release from HEK/TLR4-MD-2-CD14 stably transfected cells. IL-8 release from HEK/TLR4-MD-2-CD14 stably transfected cells induced with serogroup B meningococcal CPS-*lpxA* polymers pre-incubated with 4 μg/ml of LL-37. IL-8 was measured by ELISA method.
(0.29 MB TIF)

Table S1 L: leucine; K: lysine; R: arginine; E: glutamic acid; D: aspartic acid; A: alanine; N: asparagine FA19: non-encapsulated *Neisseria gonorrhea* MIC: minimal inhibitory concentration. NA: not available.
(0.04 MB DOC)
