# Supplementary figures and images for: The Human Host Defense Peptide LL-37 Interacts with Neisseria meningitidis Capsular Polysaccharides and Inhibits Inflammatory Mediators Release
Source: PLoS One. 2010 Oct 26;5(10):e13627. doi: 10.1371/journal.pone.0013627 (PMC2964311; doi:10.1371/journal.pone.0013627)

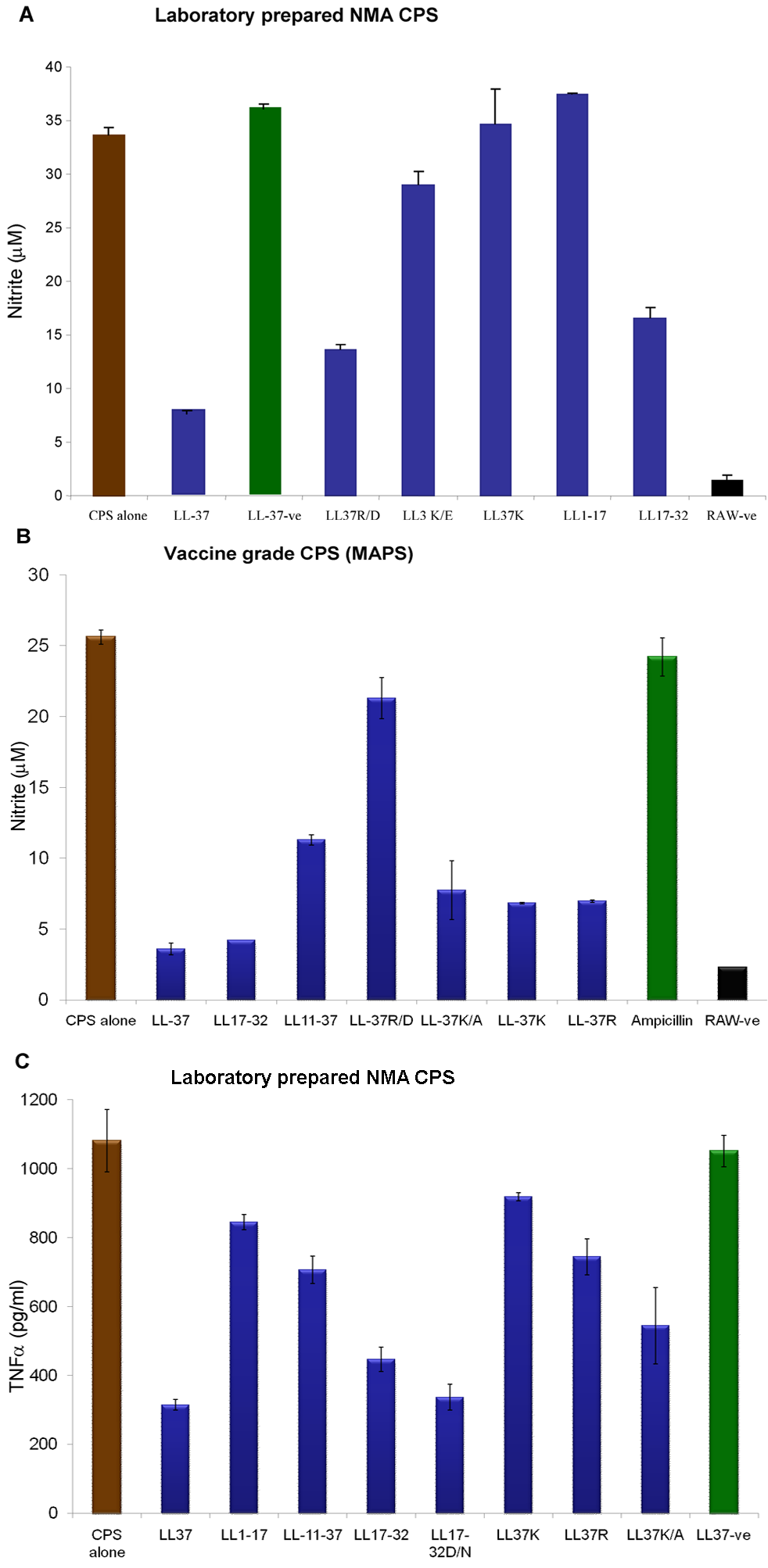

Supplement: Figure S1 — Synthetic LL-37 analogs interact with a vaccine grade meningococcal CPS. Murine RAW 264 macrophages stimulated with (A) laboratory prepared serogroup A meningococcal CPS polymers or (B) a vaccine grade serogroup A meningococcal CPS 20 μg /ml (MAPS) pre-incubated with or without 4 μg/ml of synthetic LL-37 analogs. Ampicillin, a non-cationic antibiotic, was used as a control. Nitric oxide release from induced murine cells was quantified by the Griess method as nitrite accumulation after 24 h of incubation at 37°C with 5% CO2. RAW-ve is unstimulated cells but 50 μl of equivalent PBS volume is added. C: TNFα release from human THP-1 cells stimulated with laboratory prepared meningococcal serogroup A CPS polymers pre-incubated with LL-37 analogs as in panel A. Error bars represent the ±SD from the mean of 4 independent wells. The results are representative of three independent experiments. * p values were calculated using Excel software student t-test in reference to CPS alone without LL-37 analogs. (6.85 MB TIF) [file pone.0013627.s001.tif]

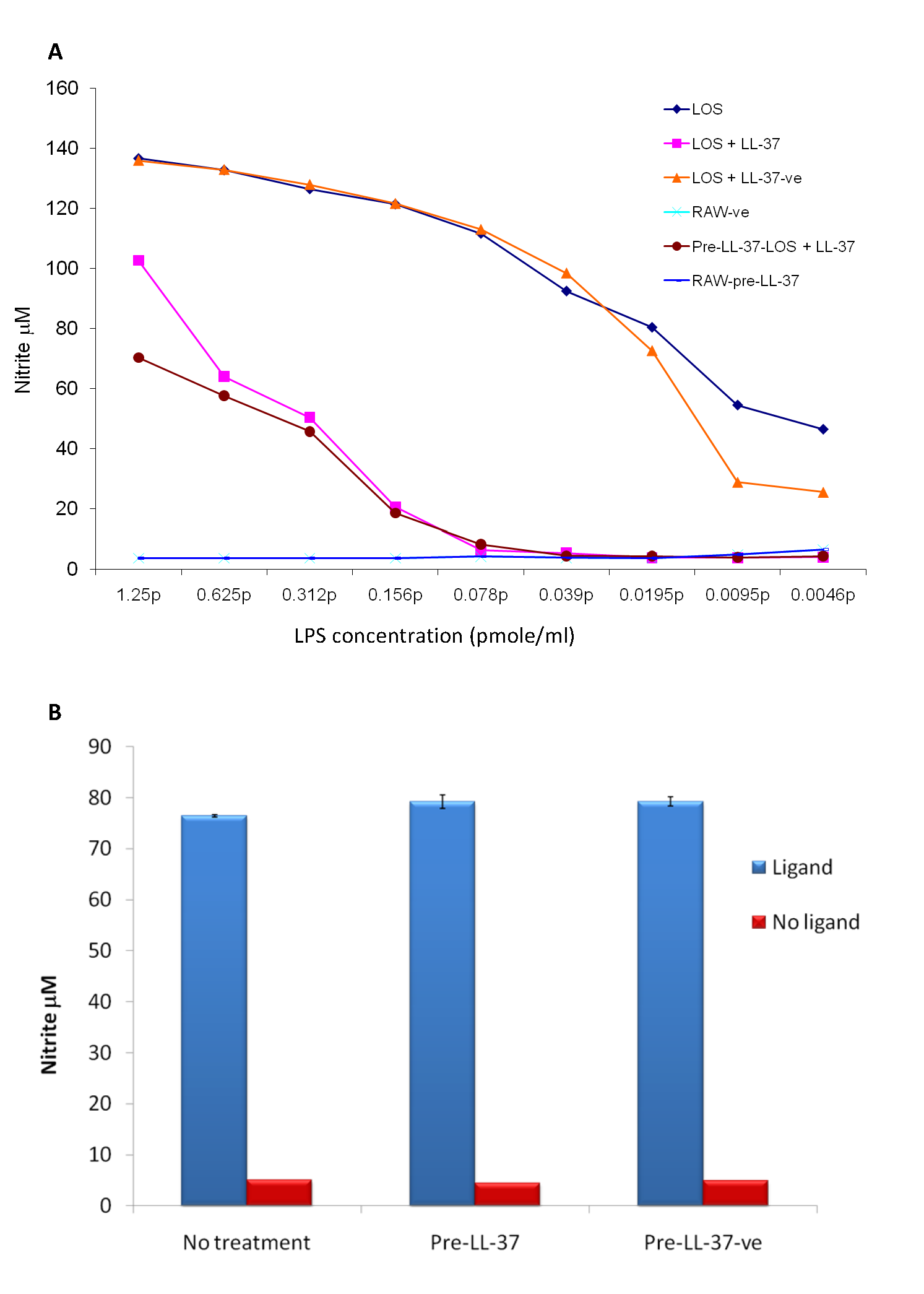

Supplement: Figure S2 — Pre-treatment of macrophages with LL-37 did not alter cellular responses. Murine macrophages RAW 264 were pre-treated with 10 μg/ml of LL-37 or its inactive analog (the negatively charged LL-37-ve) for 30 min, followed by washing to remove LL-37 prior to stimulation with meningococcal LOS doses (A) or CPS-lpxA dose of 50 μg/ml (B). Nitric oxide release was measured as nitrite accumulation in supernatants and quantified by the Greiss method. (0.60 MB TIF) [file pone.0013627.s002.tif]

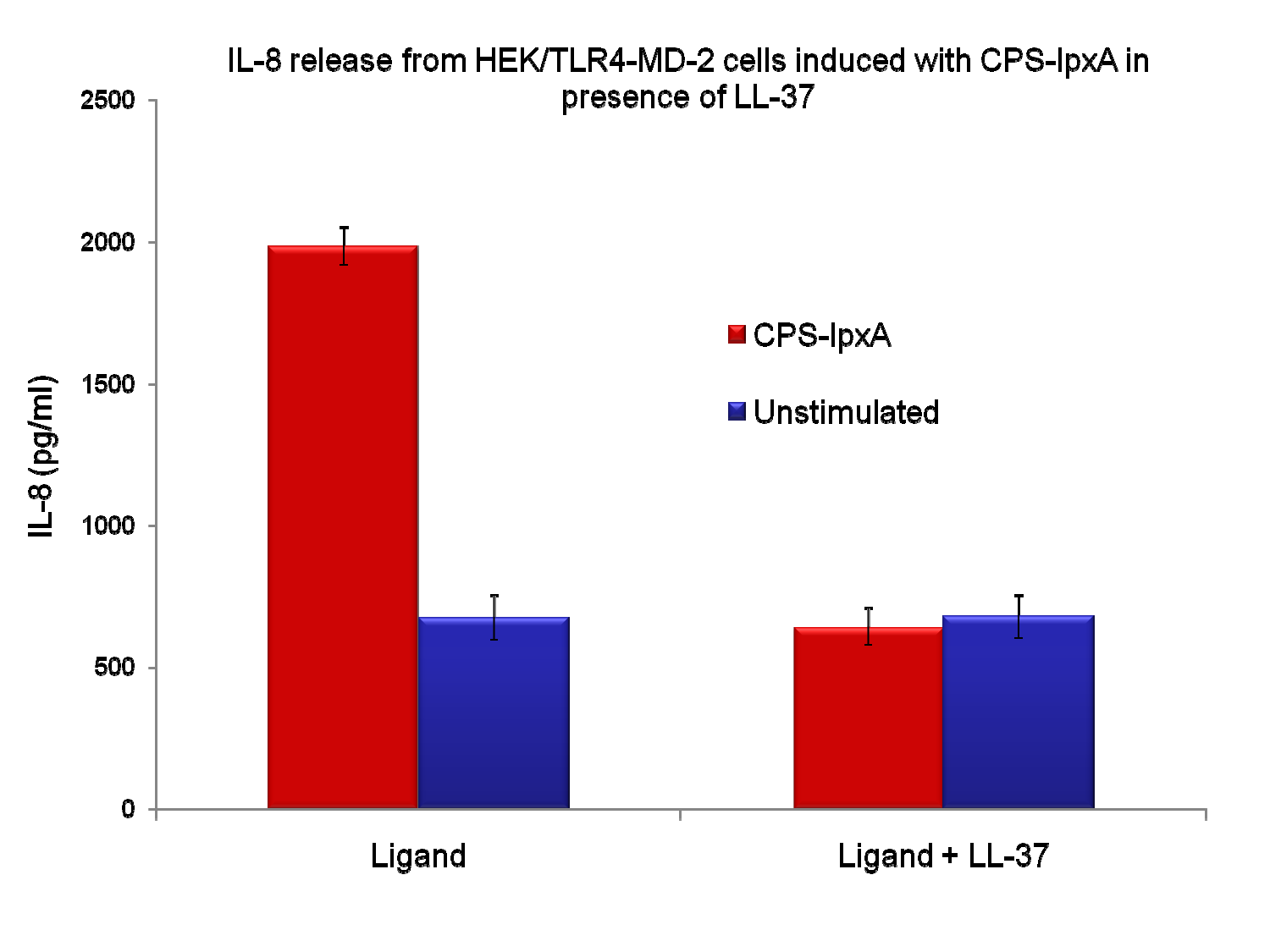

Supplement: Figure S3 — LL-37 interacts with meningococcal CPS and inhibits IL-8 release from HEK/TLR4-MD-2-CD14 stably transfected cells. IL-8 release from HEK/TLR4-MD-2-CD14 stably transfected cells induced with serogroup B meningococcal CPS-lpxA polymers pre-incubated with 4 μg/ml of LL-37. IL-8 was measured by ELISA method. (0.29 MB TIF) [file pone.0013627.s003.tif]
