# Supplementary material for: The Human Host Defense Peptide LL-37 Interacts with Neisseria meningitidis Capsular Polysaccharides and Inhibits Inflammatory Mediators Release
Source: PLoS One. 2010 Oct 26;5(10):e13627. doi: 10.1371/journal.pone.0013627 (PMC2964311; doi:10.1371/journal.pone.0013627)
Supplement: Table S1 — L: leucine; K: lysine; R: arginine; E: glutamic acid; D: aspartic acid; A: alanine; N: asparagine FA19: non-encapsulated Neisseria gonorrhea MIC: minimal inhibitory concentration. NA: not available. (0.04 MB DOC) [file pone.0013627.s004.doc]

**Table S1: LL-37 analogs, antibacterial activity and charge distribution**

**Inactive domain** Active domain

|  | MIC | LL 1-16 | LL 1-16 | LL 17-37 | LL 17-37 | Total | Total |
| --- | --- | --- | --- | --- | --- | --- | --- |
|  | µg/ml (FA19) | Pos+ve charges | Neg-ve charges | Pos+ve charges | Neg-ve charges | Pos+ve  charges | Neg-ve  charges |
| LL-37 | 7.8 | 5+ve  1R 4K | 3-ve  1D 2E | 6+ve  4R 2K | 2-ve  1D 1E | 11+ve  5R 6K | 5-ve  2D 3E |
| LL-37  R/D-K/E | >1000 | None | 8-ve  2D 6E | None | 8-ve  5D 3E | None | 16-ve  7D 9E |
| LL-37 R/D | 125 | 4+ve  0R 4K | 4-ve  2D 2E | 2+ve  0R 2K | 6-ve  5D 1E | 6+ve  0R 6K | 10-ve  7D 3E |
| LL-37 K/E | >1000 | 1+ve  1R 0K | 7-ve  1D 6E | 4+ve  4R 0K | 4-ve  1D 3E | 5+ve  5R 0K | 11-ve  2D 9E |
| LL-37 K | 7.8 | 8+ve  0R 8K | None | 8+ve  0R 8K | None | 16+ve  0R 16K | None |
| LL-37 R | 15.6 | 8+ve  8R 0K | None | 8+ve  8R 0K | None | 16+ve  16R 0K | None |
| LL-37 K/A | >1000 | 1+ve  1R 0K | 3-ve  1D 2E | 4+ve  4R 0K | 2-ve  1D 1E | 5+ve  5R 0K | 5-ve  2D 3E |
| LL-37 K/L | >1000 | 1+ve  1R 0K | 3-ve  1D 2E | 4+ve  4R 0K | 2-ve  1D 1E | 5+ve  5R 0K | 5-ve  2D 3E |
| LL-1-17 | >1000 | 5+ve  1R 4K | 3-ve  1D 2E | NA | NA | 5+ve  1R 4K | 3-ve  1D 2E |
| LL-11-37 | 7.8 | 2+ve  0R 2K | 2-ve  0D 2E | 6+ve  4R 2K | 2-ve  1D 1E | 8+ve  4R 4K | 4-ve  1D 3E |
| LL-17-32 | 3.9 | NA | NA | 5+ve  3R 2K | 1-ve  1D 0E | 5+ve  3R 2K | 1-ve  1D 0E |
| LL-17-32 D/N | 3.9 | NA | NA | 5+ve  3R 2K | None | 5+ve  3R 2K | None |
| C12LL-17-32 D/N | 3.9 | NA | NA | 5+ve  3R 2K | None | 5+ve  3R 2K | None |

L: leucine; K: lysine; R: arginine; E: glutamic acid; D: aspartic acid; A: alanine; N: asparagine

FA19: non-encapsulated *Neisseria gonorrhea*

MIC: minimal inhibitory concentration. NA: not available.
